# Salicylic Acid Improves the Constitutive Freezing Tolerance of Potato as Revealed by Transcriptomics and Metabolomics Analyses

**DOI:** 10.3390/ijms24010609

**Published:** 2022-12-29

**Authors:** Lin Chen, Feiyan Zhou, Ye Chen, Yongqi Fan, Kangkang Zhang, Qing Liu, Wei Tu, Fujing Jiang, Guangcun Li, Hongbo Zhao, Botao Song

**Affiliations:** 1 Key Laboratory of Biology and Genetic Improvement of Tuber and Root Crop, Ministry of Agriculture and Rural Affairs, South China Agricultural University, Guangzhou 510642, China; 2 Lingnan Guangdong Laboratory of Modern Agriculture, South China Agricultural University, Guangzhou 510642, China; 3 Key Laboratory of Biology and Genetic Improvement of Horticultural Crops (South China), Ministry of Agriculture and Rural Affairs, College of Horticulture, South China Agricultural University, Guangzhou 510642, China; 4 Key Laboratory of Horticultural Plant Biology, Ministry of Education, Huazhong Agricultural University, Wuhan 430070, China; 5 Key Laboratory of Potato Biology and Biotechnology, Ministry of Agriculture and Rural Affairs, College of Horticulture and Forestry Science, Huazhong Agricultural University, Wuhan 430070, China

**Keywords:** *Solanum acaule*, *Solanum tuberosum*, acclimated freezing tolerance, CBF, *HSFC1*

## Abstract

Freezing severely impacts potato production. Deciphering the pathways and metabolites that regulate the freezing tolerance of potato is useful in cultivation and breeding for hardiness. In the present study, *Solanum acaule* was identified to be more freezing tolerant than *S. tuberosum*. Furthermore, the two genotypes before/after exposure to 4 °C for 7 d with additional −1 °C for 12 h were analysed by RNA-seq and metabolomics, and the results were compared with the previous −1 °C for 12 h. The results showed that *S. acaule* activated numerous genes that differed from those of *S. tuberosum*. Among the genes, five pathways, such as the hormone signalling pathway, which includes salicylic acid, were enriched. Further metabolomics analysis showed that the content of salicylic acid was improved in *S. acaule* in response to −1 °C for 12 h. Moreover, exogenous application of 0.1 mM salicylic acid to potato was shown to improve constitutive freezing tolerance and increase the expression of *HSFC1*. Following transcriptome and metabolome analyses, it was documented that the content of SA that increased in freezing-tolerant *S. acaule* after exposure to cold condition, associated with the SA signalling pathway, enhanced potato freezing tolerance, probably through *HSFC1*.

## 1. Introduction

Cold is one of the main environmental factors limiting the geographical distribution and productivity of plants [1]. Potato (*Solanum tuberosum* L.) is the fourth most important food crop. The major potato cultivation regions are in the northern temperate zone and tropical highlands [2]. Freezing stress is a major constraint for potato production in these regions. However, potato varieties are sensitive to low temperatures and unable to cold-acclimate [3]. Even brief exposure to freezing stress can substantially reduce potato production, let alone hard frosts, which can completely devastate potato plants [4]. Therefore, unravelling how potatoes defend themselves against freezing-stress-induced impartment is not only a crucial fundamental issue but also is important for food security and agricultural sustainability.

Although potato varieties derived from *S. tuberosum* are freezing sensitive, some wild potato species, such as *S. acaule* and *S. commersonii*, exhibit freezing tolerance far superior to that of cultivated species [5,6]. Thus, they have the potential to be utilized as genetic donors to increase the cold hardiness of potato varieties. *S. acaule* is known to have a high level of frost hardiness and moderate cold acclimation capacity [7], and it is valuable to introgress freezing tolerance traits of *S. acaule* into cultivated potato. However, freezing tolerance is regulated by multiple genes, and potato inheritance patterns are complex [8]. Deciphering the pathways and genes underlying freezing tolerance is difficult through quantitative trait locus mapping and further cloning.

Omics approaches have promoted progress in deciphering plant stress-responsive mechanisms and pathways during the last decade [9]. Transcriptomics, which can be used to segregate cold-responsive genes, is a useful tool to decipher freezing tolerance genes. In our previous research, Chen et al. [10] successfully identified the mitogen-activated protein kinase MKK2, which positively regulates the constitutive freezing tolerance of *S. acaule*. Chen et al. [11] identified a calcineurin B-like gene that positively regulates constitutive and acclimated freezing tolerance in *S. acaule*, *S. commersonii*, and *S. malmeanum*. However, thousands of genes are cold-responsive in potato after exposure to cold conditions, which makes it difficult to identify the key freezing-tolerant genes. Integrated omics approaches, such as transcriptomics and metabolomics, are useful tools in deciphering the underlying pathways and genes contributing to the freezing tolerance of plants [12]. In our previous research, transcriptome and metabolome analyses were carried out to successfully identify the freezing-tolerance gene, arginine decarboxylase gene 1, and its associated metabolite, putrescine, which regulates the cold-acclimated freezing- tolerance of *S. acaule* [13]. This suggests that the combination of transcriptomics and metabolomics analyses is an efficient tool to identify the freezing-tolerance pathway of potato.

Plants implement various endogenous strategies, such as plant hormone biosynthesis, to withstand abiotic stresses. Plant hormones are a driving force for modulating abiotic stress responses, plant growth, and developmental mechanisms [14]. Some hormones are known to play important roles in the plant response to cold stress, such as salicylic acid (SA). SA is synthesized from either precursor isochorismic acid by the isochorismate pathway or the precursor benzoic acid by the phenylalanine ammonium lyase pathway [15]. SA is well-known for its role in mediating defence against pathogens [16]. Numerous studies support that SA also plays important roles in the freezing tolerance of plants, such as spinach [16] and wheat [17]. Mora-Herrera et al. [18] indicated that SA could improve the freezing tolerance of potato. However, the freezing tolerance of potato consists of constitutive freezing tolerance and cold acclimation capacity. Constitutive freezing tolerance indicates the ability of plants to survive under freezing temperatures when they grow under normal conditions without pretreatment under nonlethal cold conditions. Cold acclimation capacity implies the capacity of plants to increase freezing tolerance with nonlethal cold temperature pretreatment. The two types of freezing tolerance are under independent genetic control in potato [19]. Both constitutive freezing tolerance and cold acclimation capacity are significantly correlated with potato frost tolerance in the field [20]. However, they are only weakly correlated with each other [19]. Since Mora-Herrera et al. [18] only analysed the effect of SA on constitutive freezing tolerance, it could only be concluded that SA could improve the constitutive freezing tolerance of potato. Whether SA has an effect or not on the acclimated freezing tolerance of potato yet, is still unknown. Meanwhile, Mora-Herrera et al. [18] indicated that SA improved the freezing tolerance of potato not via H_2_O_2_. The functional mechanism of SA in improving the freezing tolerance of potato is still unknown.

CBFs (C-repeat binding factors) play central roles in regulating plant freezing tolerance [21]. After exposure to cold stress, CBF proteins bind to CRT/DRE cis-elements in the promoters of cold-regulated (COR) genes and activate their transcription [22]. Since CBF only accounts for 10–15% of *COR* genes, other transcription factors, in addition to *CBFs*, also play important roles in regulating freezing tolerance, such as those known as the “first-wave” transcription factors, whose expression patterns are similar to those of *CBFs* in response to cold stimuli. These transcription factors regulate freezing tolerance independent of the *CBF* regulatory module [23]. Among them, *HSFC1*, *ZAT10,* and *CZF1* regulate the expression of *CORs* without inducing the expression of *CBFs*.

The role of SA in regulating the expression of *CBF* is species dependent. SA has been shown to alleviate freezing damage to peach floral organs and enhance freezing tolerance by upregulating the expression of *CBF* genes [24]. However, SA increases the freezing tolerance of watermelon but negatively regulates the CBF-dependent cold-responsive pathway [25]. Wang et al. [26] indicated that SA has a slight but not significant effect on CBF expression in winter wheat. Therefore, the effect of SA on *CBF* expression in the freezing tolerance of potato needs to be deciphered.

To unravel the potential pathways and metabolites that regulate the freezing tolerance of *S. acaule*, freezing-tolerant *S. acaule* (aca) and freezing-sensitive *S. tuberosum* (tub) were used as models in the present research. Aca was identified to be more constitutive and acclimated freezing tolerant that tub. We further compared transcriptomic and metabolomic differences between aca and tub in response to 4 °C for 7 d with additional −1 °C for 12 h. The results were also compared with the previous −1 °C for 12 h. The result showed that, compared with tub, aca especially enriched hormone signalling pathways, including the SA signalling pathway, in response to both cold conditions. Further metabolomic analysis showed that the content of SA was increased in aca only after exposure to −1 °C for 12 h. Exogenous application of SA in potato showed that salicylic acid could improve the constitutive freezing tolerance of potato. To decipher whether exogenous application of SA on potato improved freezing tolerance by enhancing the expression of *CBF* or other genes, further RNA-seq analysis was carried out on tub with exogenous application of SA and distilled water. The results showed that exogenous application of SA increased *HSFC1* expression compared with the mock, which suggests that SA possibly increases freezing tolerance via non-*CBF* transcription factors, such as *HSFC1*.

## 2. Results

### 2.1. Aca and Tub Showed Distinct Freezing Tolerance

**Figure 1:**
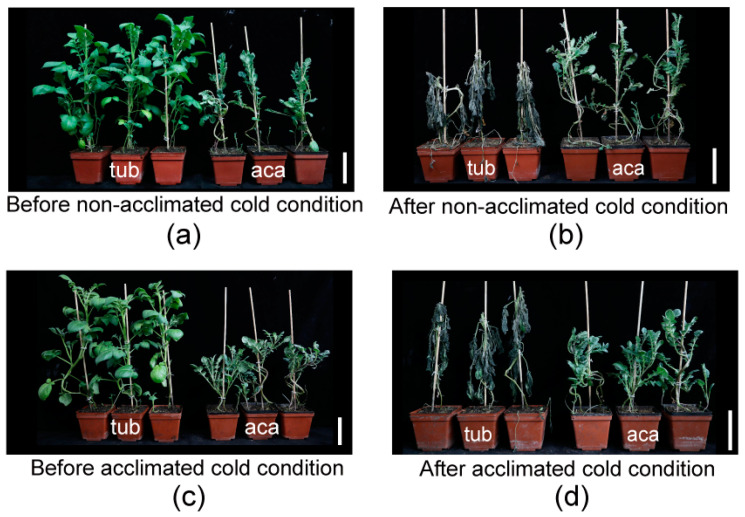
Freezing tolerance measurements of freezing-tolerant aca and freezing-sensitive tub. (**a**) Aca and tub plantlets before non-acclimated cold condition. (**b**) Plantlets of (**a**) subjected to −3.0 °C for six hours, followed by two days at appropriate temperature (22 °C) for recovery (after non-cold-acclimated cold condition). (**c**) Aca and tub plantlets that have been cold-acclimated at 4 °C for 7 d (before acclimated cold condition). (**d**) Plantlets of (**c**) subjected to −3.5 °C for six hours, followed by two days at appropriate temperature for recovery (after acclimated cold condition). Three independent biological replicates were carried out, and similar phenotypes were obtained. The bar represents 10 cm.

In our previous research, aca was more freezing tolerant than tub, with a significantly lower LT_50_ (semilethal temperature) determined by the electrolyte leakage of excised leaflets [13]. To determine whether aca suffered from less freezing damage than tub, the freezing damage of the whole plantlets before/after being transferred to cold conditions was evaluated. Three independent biological replicates were carried out, and similar phenotypes were obtained. Constitutive freezing-tolerance phenotyping was assessed on the plantlets after being subjected to −3.0 °C for six hours, followed by two days at appropriate temperature (22 °C) for recovery (non-acclimated cold condition). Acclimated freezing-tolerance phenotyping was assessed on the plantlets after cold acclimation at 4 °C for 7 d and transfer to −3.5 °C for six hours, followed by two days at appropriate temperature for recovery (acclimated cold condition). The temperatures of −3.0 °C for constitutive freezing-tolerance phenotyping and −3.5 °C for acclimated freezing-tolerance phenotyping were applied, since the freezing-damage phenotype of the potato plantlets was obvious after treatment under the two temperatures [11]. The temperature of acclimated freezing-tolerance phenotyping (−3.5 °C) was slightly less than the one of constitutive freezing-tolerance phenotyping (−3.0 °C) because potato has an acclimation capacity. As shown in Figure 1a,b, tub derived from the cultivar grew much more vigorously, with larger leaves, higher plant height, and thicker stems than aca derived from wild species at the same growth stage (Table 1). However, after exposure to non-acclimated (Figure 1c) and acclimated cold conditions (Figure 1d), tub suffered from more severe damage compared to aca. This indicated that aca was more constitutive and acclimated freezing tolerant than tub. To decipher the underlying mechanism of the distinct freezing tolerance of aca and tub, RNA-seq was further performed.

### 2.2. Acquisition of High-Quality RNA-Seq Data for Further Analysis

The RNA-seq data of aca and tub in response to 4 °C for 7 d with additional −1 °C for 12 h were generated as a part of the present study, and the data for aca and tub in response to −1 °C for 12 h were obtained from the National Center for Biotechnology Information, accession SRP076322 (the data from our previous study [13]). Leaf samples of four-week-old plantlets were taken and used for RNA sequencing. Five independent biological replicates of each treatment were collected, three of which were randomly selected and used for RNA-seq analysis. Each biological replicate was pooled by three technical replicates. In each technical replicate, five leaves of a plantlet were used. High-quality RNA-seq data, referred to as those containing high percentages of clean reads and Q30 (base accuracy of more than 99.9%), as well as high mapping rates to the potato reference genome, were derived from aca and tub upon 4 °C for 7 d with additional −1 °C for 12 h. In total, 62.8 million raw reads were obtained, with 10.5 million raw reads per sample (Table S2). The clean reads accounted for an average of 92.5% of the raw reads after trimming. In line with this, the average percentage of Q30 of the clean reads was 94.5%. Additionally, the clean reads exhibited high mapping rates (average mapping rate of 81.3%) when they were mapped to the potato reference genome (http://solanaceae.plantbiology.msu.edu/pgsc_download.shtml, accessed on 29 December 2022). The high ratio of clean reads and high Q30 and mapping rates indicated the high quality of the RNA-seq data in the present study, suggesting that the RNA-seq data were suitable for further analysis.

To verify the accuracy of the RNA-seq data, qPCR was carried out to detect the expression of eight randomly selected genes. The expression patterns detected by qPCR and RNA-seq were similar (R^2^ of the log2-fold change was 0.8291, Supplementary Figure S1). These results suggested that the RNA-seq data correctly captured the expression patterns and were deemed appropriate for further analysis.

### 2.3. Differentially Expressed Genes (DEGs) between Aca and Tub under the Same Conditions

**Figure 2:**
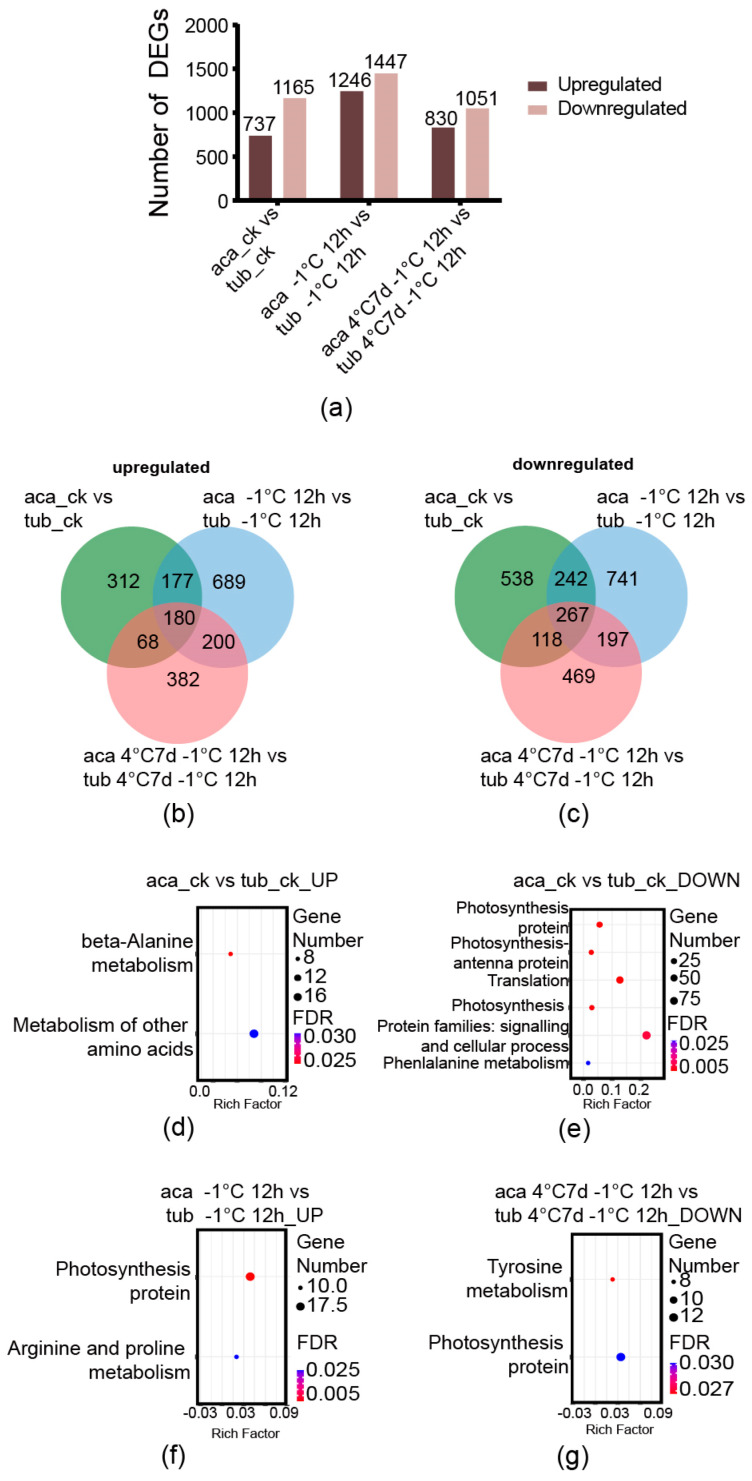
Venn diagrams of cold-responsive DEGs derived from the comparison between aca and tub under the same conditions, as well as the enriched KEGG pathways. (**a**) The numbers of DEGs in the comparison between aca and tub at appropriate temperature (aca_ck vs. tub_ck), −1 °C for 12 d (aca −1 °C 12 d vs. tub −1 °C 12 d), and 4 °C for 7 d with additional −1 °C for 12 d (aca 4 °C 7 d −1 °C 12 d vs. tub 4 °C 7 d −1 °C 12 d). (**b**) Venn diagram of upregulated DEGs from the comparison between aca and tub at appropriate temperature, −1 °C for 12 d, and 4 °C for 7 d with additional −1 °C for 12 d. (**c**) Venn diagram of downregulated DEGs from the comparison between aca and tub at appropriate temperature, −1 °C for 12 d, and 4 °C for 7 d with additional −1 °C for 12 d. (**d**) Enriched KEGG pathways of upregulated DEGs from the comparison between aca and tub at appropriate temperature. (**e**) Enriched KEGG pathways of downregulated DEGs from the comparison between aca and tub at appropriate temperature. (**f**) Enriched KEGG pathways of upregulated DEGs from the comparison between aca and tub at −1 °C for 12 d. (**g**) Enriched KEGG pathways of downregulated DEGs from the comparison between aca and tub at 4 °C for 7 d with additional −1 °C for 12 d.

The number of DEGs was analysed first. The genes derived from the comparisons between aca and tub under the same conditions with an absolute value of log-2 (fold change with FPKM) ≥ 1 and false discovery rate (FDR) ≤ 0.001 were used as the DEGs in this analysis. The numbers of upregulated DEGs derived from the comparisons between aca and tub under appropriate temperature (22 °C), –1 °C for 12 h, and 4 °C for 7 d with additional –1 °C for 12 h were 737, 1246, and 830, respectively. The numbers of downregulated DEGs derived from the same comparisons were 1165, 1447, and 1051, respectively (Figure 2a). The DEGs of the comparison between aca and tub under appropriate temperature were thought to represent the difference in their genetic backgrounds. When the genotypes were transferred into cold conditions, the numbers of DEGs in the comparison between aca and tub increased. Notably, the numbers of both upregulated and downregulated DEGs of the comparison between aca and tub under –1 °C for 12 h were the largest. This might suggest that, when aca and tub were transferred into –1 °C for 12 h, numerous genes were changed for potato to adapt to the cold condition. The numbers of both upregulated and downregulated DEGs in the comparison between aca and tub under 4 °C for 7 d with additional –1 °C for 12 h were less than the ones under –1 °C for 12 h. This implied that, with prolonged cold treatment, fewer genes were activated. To decipher the difference among the DEGs of aca and tub under appropriate temperature, –1 °C for 12 h and 4 °C for 7 d with followed –1 °C for 12 h, Venn diagrams were constructed.

In the upregulated DEGs, there were 180 common genes among the comparisons between aca and tub under appropriate temperature, –1 °C for 12 h and 4 °C for 7 d with additional –1 °C for 12 h (Figure 2b). Additionally, 689 genes were only activated in the comparison of aca and tub under –1 °C for 12 h, and 382 genes were only activated in the comparison of aca and tub under 4 °C for 7 d with additional –1 °C for 12 h. Moreover, in the downregulated DEGs, 267 common genes were identified among the comparisons between aca and tub under appropriate temperature, –1 °C for 12 h and 4 °C for 7 d with additional –1 °C for 12 h (Figure 2c). Additionally, 741 genes were only activated in the comparison of aca and tub under –1 °C for 12 h, and 469 genes were only activated in the comparison of aca and tub under 4 °C for 7 d with additional –1 °C for 12 h. Numerous DEGs seem to only be activated in the comparison of aca and tub under –1 °C for 12 h or 4 °C for 7 d with additional –1 °C for 12 h. Aca activated numerous different genes compared with tub after exposure to cold conditions. To provide insight into the DEGs, KEGG pathway enrichment analysis was carried out.

An FDR-corrected *p*-value ≤ 0.05 was used as a cut-off value for the significance of KEGG enrichment analyses. KEGG pathways were enriched only in the upregulated and downregulated DEGs in the comparison between aca and tub under appropriate temperature. Results showed upregulated DEGs in the comparison between aca and tub at –1 °C for 12 h, and downregulated DEGs in the comparison between aca and tub 4 °C for 7 d with additional –1 °C for 12 h. In the upregulated and downregulated DEGs between aca and tub under appropriate temperature, some amino acid-related pathways were enriched, such as “beta-alanine metabolism”, “metabolism of other amino acids”, and “phenylalanine metabolism” (Figure 2d,e). These results implied that aca and tub have some differences in amino acid metabolism under appropriate temperature.

Furthermore, in the enriched KEGG pathways of upregulated DEGs in the comparison between aca and tub under –1 °C for 12 h, the pathway “arginine and proline metabolism” was enriched (Figure 2f). The products of the pathway related to the DEGs included agmatine, 4-amino butanoate, spermidine, putrescine, glutamate, and 1-pynoline 5-carboxylate (Supplementary Figure S2). This suggested that the metabolites might be related to the freezing tolerance of aca. In the enriched KEGG pathways of downregulated DEGs in the comparison between aca and tub under 4 °C for 7 d with additional –1 °C for 12 h, the pathway “tyrosine metabolism” was enriched (Figure 2g). The products of the pathway related to the DEGs comprised L-phenylalanine, phenylpyruvate, phenyl-ethylamine, phenyl-acetaldehyde, 2-Hydroxy-phenylacetate, 2-hydroxy-3-phenylpropanote, and trans-cinnamate (Supplementary Figure S3). These results suggested that these metabolites might be related to tub in response to 4 °C for 7 d with additional –1 °C for 12 h.

### 2.4. The Gene Sets of Aca in Response to Cold Conditions

**Figure 3:**
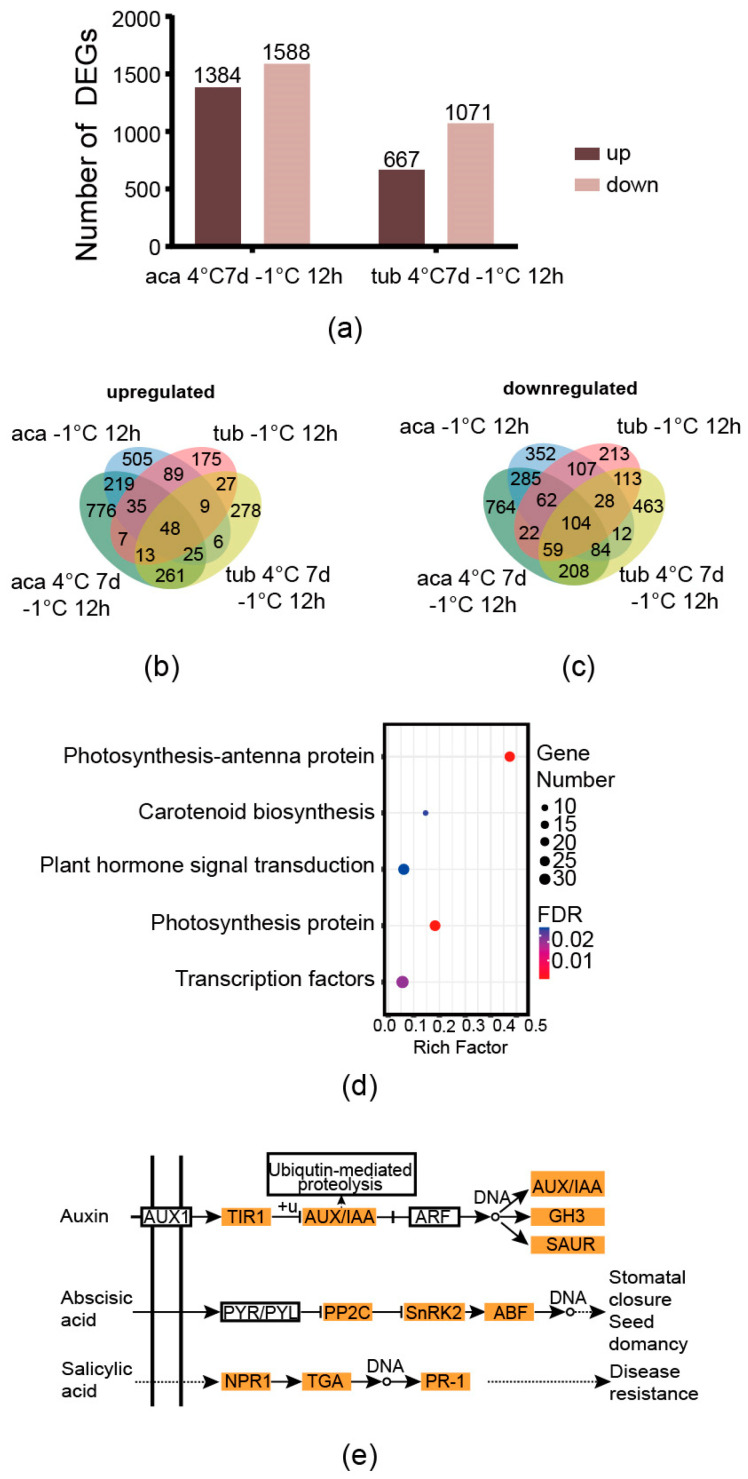
Venn diagram of cold-responsive DEGs of aca and tub, as well as enriched pathways of aca in response to cold conditions. (**a**) The numbers of DEGs of aca and tub exposed to 4 °C for 7 d with additional −1 °C for 12 d compared to the control condition (22 °C). (**b**) Venn diagram of upregulated DEGs of aca and tub after exposure to cold conditions compared to the control condition. (**c**) Venn diagram of downregulated DEGs of aca and tub after exposure to cold conditions compared to the control condition. (**d**) Enriched KEGG pathways of the overlapping DEGs of aca in response to both types of cold conditions compared with the control condition. (**e**) The DEGs in the KEGG pathway “plant hormone signal transduction” as indicated in (**d**). AUX1, auxin influx carrier 1; TIR1, transport inhibitor response 1; AUX, auxin-responsive protein; IAA, indoleacetic acid-induced protein; ARF, auxin response factor; GH3, auxin-responsive Gretchen Hagen 3 gene family; SAUR, small auxin upregulated RNA protein; PYR, pyrabactin resistance protein; PYL, pyrabactin resistance-like protein; PP2C, protein phosphatase 2C; SnRK2, sucrose non-fermenting-1-related protein kinase 2; ABF, abscisic acid responsive element binding factor; NPR1, nonexpresser of pathogenesis-related genes 1; TGA, TGACG sequence-specific binding protein; PR-1, pathogenesis-related protein 1.

The genes derived from the comparisons between aca/tub after exposure to cold conditions compared to the those under appropriate temperature with an absolute value of the log-2 (fold change with FPKM) ≥1 and false discovery rate (FDR) ≤0.001 were used as the DEGs in this analysis. After exposure to 4 °C for 7 d with additional –1 °C for 12 h, there were 1384 and 667 upregulated DEGs in aca and tub, respectively. Moreover, there were 1588 and 1071 downregulated DEGs in aca and tub, respectively. There were more upregulated and downregulated genes in freezing-tolerant aca than in freezing-sensitive tub in response to 4 °C for 7 d with additional –1 °C for 12 h (Figure 3a). The same trend was observed in aca and tub in response to –1 °C for 12 h [13]. This indicated that aca recruited more genes in response to freezing conditions than tub.

There was a large proportion of differentially-regulated genes and some commonly-regulated genes of aca in response to –1 °C for 12 h and 4 °C for 7 d with additional –1 °C for 12 h. With regard to upregulated genes, there were 53.0%, 16.4%, and 30.6% of acclimated-freezing-condition-specific-responsive genes (the genes that activated only in 4 °C for 7 d with additional –1 °C for 12 h), overlapping genes, and non-acclimated-freezing-condition-specific-responsive genes (the genes that activated only in –1 °C for 12 h), respectively (taking the upregulated DEGs of aca at 4 °C for 7 d with additional –1 °C for 12 h and aca at –1 °C for 12 h compared with the control condition as 100%, Figure 3b). Regarding downregulated genes, the same trend was observed, with 51.7%, 25.0%, and 23.3%, respectively (regarding the downregulated DEGs of aca 4 °C for 7 d with followed –1 °C for 12 h and aca –1 °C for 12 h as 100%, Figure 3c). This suggested that a large proportion of cold-responsive genes that aca evolved in response to –1 °C for 12 h and 4 °C for 7 d with additional –1 °C for 12 h were different. Freezing-tolerant aca activated different gene sets in response to different cold stimuli. This might support that acclimation capacity is not related to constitutive freezing tolerance in potato [19]. However, aca still evolved some common genes in response to the two types of cold conditions.

Since the genes that regulate freezing tolerance might be a common cold-responsive gene set of aca in response to both –1 °C for 12 h and 4 °C for 7 d with additional –1 °C for 12 h, the gene set was further analysed. As shown in Figure 3b,c, 219 genes were upregulated in the freezing-tolerant aca in response to both types of cold conditions but not in the freezing-sensitive tub. In addition, 285 cold-responsive genes were downregulated in aca but not in tub. It was speculated that the genes might be related to freezing tolerance in aca. KEGG and GO enrichment analyses were further carried out on the genes to provide more information. An FDR-corrected *p*-value ≤ 0.05 was used as a cut-off value for the significance of GO and KEGG enrichment analyses. As shown in Figure 3d, five KEGG pathways, including photosynthesis-antenna protein, carotenoid biosynthesis, plant hormone signal transduction, photosynthesis proteins, and transcription factors, were enriched in the gene set. The enriched GO pathways included the metabolic process, the protein metabolic process, the cellular protein metabolic process, and translation (Supplementary Figure S4). Two photosynthesis-related KEGG pathways were enriched in aca in response to cold conditions. This implied that aca might adjust its photosynthetic capacity after exposure to cold conditions to alleviate the negative effect of photoinhibition. Carotenoids are known to play a role in photoprotection in plants, such as *Buxus sempervirens* L.; during winter acclimation [27]. Transcription factors, such as *CBF*, play a central role in plants in response to freezing stress. This result indicated that aca activated some transcription factors after exposure to cold conditions. We further analysed the genes of the KEGG pathway plant hormone signal transduction, since hormones play important roles in the signal transduction of freezing tolerance. As shown in Figure 3e, the expression profiles of most of the genes in the auxin, abscisic acid, and salicylic acid signalling pathways were changed. Since abscisic acid and salicylic acid were related to plants in response to abiotic stresses, the genes in the two pathways were all upregulated, which suggested that the two types of hormones might be related to the freezing tolerance of potato.

### 2.5. Metabolic Profiling of Aca and Tub before/after Exposure to Cold Conditions

**Figure 4:**
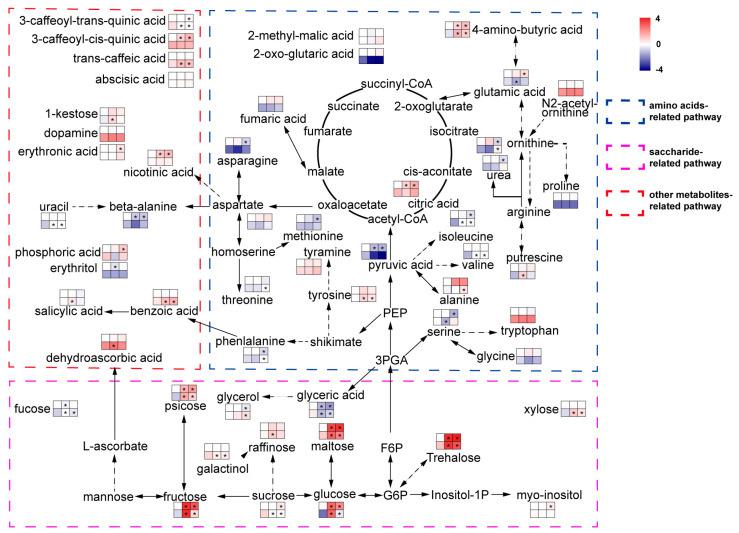
Metabolites of aca and tub after exposure to cold conditions. Metabolites were classified into three groups according to KEGG annotation. The top left square to right square indicates tub at appropriate temperature, tub upon −1 °C for 12 d, and tub upon acclimated cold condition 4 °C for 7 d with additional −1 °C for 12 h. The bottom left square to right square indicates aca with the same treatments as tub. Colour represents the log2-fold change in the metabolite content by treatments compared with the one of tub at appropriate temperature. The content of tub at appropriate temperature was set to one. Asterisks indicate the metabolite that varied significantly in response to freezing stress/control in the same genotype, and an independent *t*-test was used with *p* ≤ 0.05. The diagram was greatly simplified, with the majority of intermediates and bypasses cancelled to accentuate the metabolites that were detected in the present research. Variation of upstream metabolite content might not be in line with downstream metabolite content in the figure because the metabolic pathway was simplified.

Leaf samples of four-week-old plantlets were taken and used for metabolomics analysis. The metabolite contents of aca and tub before and after exposure to 4 °C for 7 d with additional −1 °C for 12 h were measured by GC-MS (metabolites except ABA and SA) and ELISA (ABA and SA) (Figure 4 and Supplementary Table S3). The data were compared with corresponding data of −1 °C 12 h from previous research [13]. Five independent biological replicates of each treatment were collected and used for metabolomics analysis. Each biological replicate was pooled by three technical replicates. In each technical replicate, five leaves of a plantlet were used. In total, 53 metabolites were detected in the present study (Figure 4 and Supplementary Table S3). The metabolisms that varied significantly in the genotype in response to freezing stress compared with the one at appropriate temperature were analysed by an independent *t*-test with *p* ≤ 0.05.

After exposure to 4 °C for 7 d with additional −1 °C for 12 h, compared with tub, for the metabolites in the category “amino acids-related pathway”, aca specifically increased the contents of many amino acids, including alanine, isoleucine, ornithine, phenylalanine, threonine, tyrosine, and valine. In the category “saccharide-related pathway”, compared with tub, aca specifically increased the content of fucose, glycerol, raffinose, and xylose, but decreased the content of galactinol and sucrose. For metabolites in the category of “other metabolite-related pathways”, aca specifically increased the content of uracil, trans-caffeic acid, and benzoic acid, but decreased the content of 3-caffeoyl-trans-quinic acid compared with tub. This indicates that aca accumulates many kinds of amino acids, sugars, and other types of metabolites in response to acclimated freezing stress.

When the genotypes were exposed to −1 °C for 12 h, with regard to the metabolites in the category of “amino acid-related pathway”, aca specifically accumulated some amino acids under −1 °C for 12 h, such as alanine, isoleucine, tyrosine, valine, and putrescine, but specifically decreased the contents of glutamic acid and serine compared with tub. With regard to the category of “saccharide-related pathway”, aca specifically increased the contents of fucose, raffinose, and xylose, but decreased the contents of galactinol and sucrose compared with tub. For metabolites in the category of “other metabolite-related pathways”, compared with tub, aca specifically increased the contents of benzoic acid, SA, uracil, trans-caffeic acid, 1-kestose, and dehydroascorbic acid, but decreased the content of 3-caffeoyl-trans-quinic acid. This indicates that aca also accumulates many kinds of amino acids, sugars, and other types of metabolites in response to −1 °C for 12 h. However, the types of amino acids, sugars, and other types of metabolites were somewhat different in aca in response to −1 °C for 12 h and 4 °C for 7 d with additional −1 °C for 12 h. The results of transcriptomics analysis also showed that aca activated different gene sets in response to different cold stimuli. This might also support that acclimation capacity was not related to constitutive freezing tolerance in potato, because potato activated different transcripts and metabolites in response to −1 °C for 12 h and 4 °C for 7 d with additional −1 °C 12 h.

Transcriptomics analysis suggested that ABA and SA signalling pathways were activated in aca in response to both −1 °C for 12 h and 4 °C for 7 d with additional −1 °C for 12 h. The ABA content was nearly unchanged in aca in response to cold conditions. However, the SA content was significantly improved in aca after exposure to −1 °C for 12 h. Although the contents of many metabolites varied, only transcripts and metabolites related to SA varied at the same time. This suggested that SA might be related to freezing tolerance by transcriptomics and metabolomics analyses.

Referring to the comparison of aca and tub under the same conditions, although the metabolomics assay did not cover any metabolites of the comparison of aca and tub under 4 °C for 7 d with followed –1 °C for 12 h, the product putrescine in the enriched KEGG pathway “arginine and proline metabolism” of the upregulated DEGs derived from the comparison between aca and tub under –1 °C for 12 h was identified. We further compared the contents of putrescine in aca and tub at –1 °C for 12 h. The content of aca at –1 °C for 12 h was 0.55 ± 0.12, which was significantly larger than that of tub with 0.28 ± 0.12 (*p* < 0.05). Putrescine was identified to regulate potato freezing tolerance in our previous research [13]. Therefore, it is intriguing to identify whether SA could also enhance freezing tolerance in potato, similar to putrescine.

### 2.6. Exogenous Application of Salicylic Acid Improved the Freezing Tolerance of Tub

**Figure 5:**
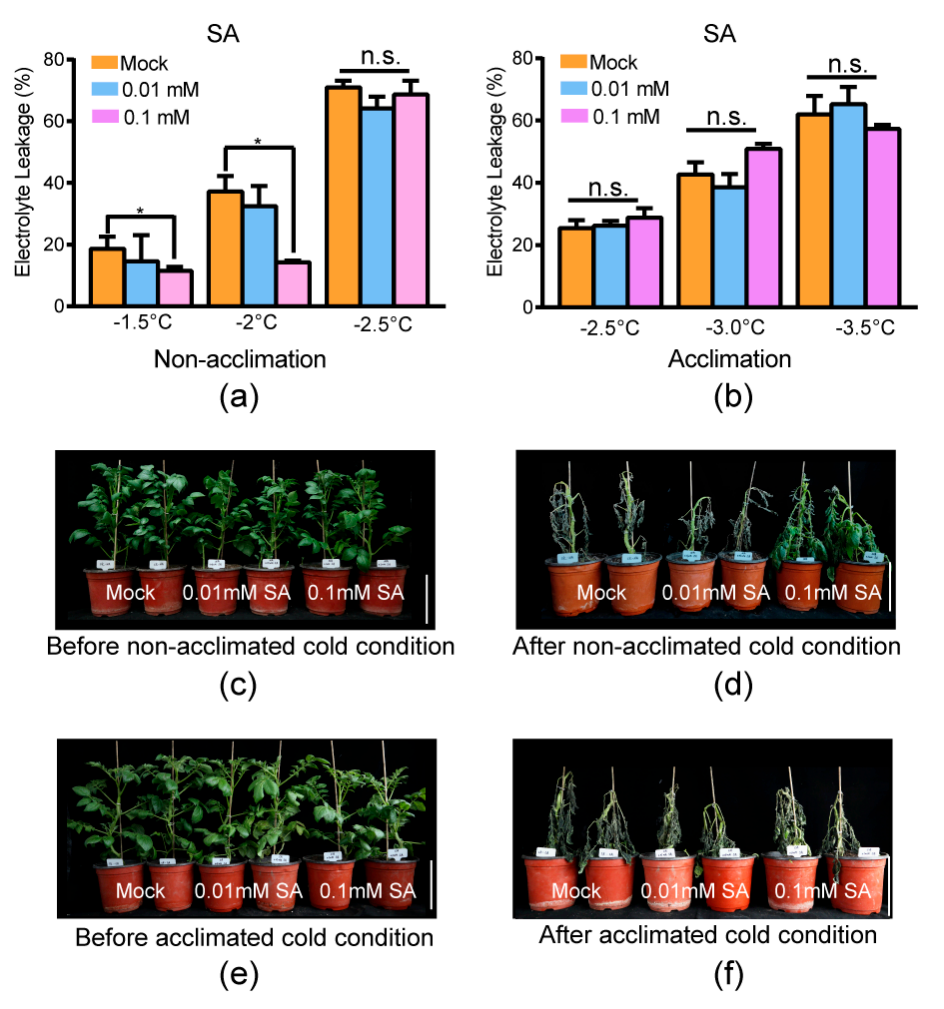
Exogenous application of 0.1 mM SA increased the constitutive freezing tolerance of potato. (**a**) Electrolyte leakage of non-cold-acclimated leaves of tub with distilled water (mock) and exogenous application of SA at the indicated temperature. Mean and S.E. from at least three independent biological replicate measurements were used. (**b**) Electrolyte leakage of cold-acclimated leaves of tub with water and exogenous application of SA at the indicated temperature. Mean and S.E. from three independent biological replicate measurements were used. Asterisks indicate significant differences between the mock and exogenous application of SA detected by Student’s *t*-test, *p* < 0.05. n.s.: no significance. (**c**) Plantlets of tub with water and exogenous application of SA before non-acclimated cold condition. (**d**) Plantlets of (**c**) subjected to −3.0 °C for six hours, followed by two days at appropriate temperature (22 °C) for recovery (after non-acclimated cold condition). (**e**) Plantlets of tub with exogenous application of SA and water after cold acclimation at 4 °C for 7 d (before acclimated cold condition). (**f**) Plantlets of (**e**) subjected to −3.5 °C for six hours, followed by two days at appropriate temperature for recovery (after acclimated cold condition). Three independent biological replicates were carried out and similar phenotypes were obtained. The bar represents 10 cm.

Two different experiments were carried out to test the effect of SA: electrolyte leakage measurement of the excised leaflets and freezing damage of the whole plantlets before/after being transferred to cold conditions. The temperatures used in the electrolyte leakage measurement of the excised leaflets (−1.5 °C/−2.0 °C/−2.5 °C for constitutive freezing-tolerance phenotyping and −2.5 °C/−3.0 °C for acclimated freezing-tolerance phenotyping) were in accordance with our previous research [11]. The temperatures of acclimated freezing-tolerance phenotyping were slightly less that the ones of constitutive freezing-tolerance phenotyping because potato has an acclimation capacity. Under non-acclimation cold conditions (without acclimation at 4 °C for 7 d), the exogenous application of 0.01 mM SA had no significant difference compared with the mock at –1.5 °C, –2.0 °C, and –2.5 °C (Figure 5a, the assay was carried out three independent times and the three independent biological replicates were used for significance analysis by independent *t*-test; significance indicated by *p* ≤ 0.05). However, exogenous application of 0.1 mM SA resulted in significantly less electrolyte leakage compared with the mock at –1.5 °C and –2.0 °C. These results indicated that exogenous application of 0.1 mM SA enhanced the constitutive freezing tolerance of potato (Figure 5a). The phenotype of whole plantlets before/after being transferred to cold conditions supported this conclusion, since plantlets with exogenous application of 0.1 mM SA suffered less freezing damage than mock plantlets (Figure 5c,d, three independent biological experiments were carried out and similar phenotypes were obtained). The exogenous application of 0.01 mM SA resulted in freezing damage similar to that in the mock treatment. This result suggested that exogenous application of 0.01 mM SA could not improve constitutive freezing tolerance (without prior acclimation). SA might improve the constitutive freezing tolerance of potato with a relatively higher concentration.

Under acclimated cold conditions (acclimated at 4 °C for 7 d), the exogenous application of both 0.1 mM and 0.01 mM SA had no significant difference compared with mock at –1.5 °C, –2.0 °C, and –2.5 °C (Figure 5b, the assay was carried out three independent times and the three independent biological replicates were used for significance analysis by independent *t*-test; significance indicated by *p* < 0.05). Meanwhile, the phenotype of whole plantlets before/after being transferred to cold conditions showed that plantlets with exogenous application of both 0.1 mM and 0.01 mM SA suffered similar freezing damage as the mock (Figure 5e,f, three independent biological experiments were carried out and similar phenotypes were obtained). This indicated that SA can only improve the constitutive freezing tolerance of potato.

### 2.7. Exogenous Application of Salicylic Acid Increased the Expression of HSFC1 but Not CBF

**Figure 6:**
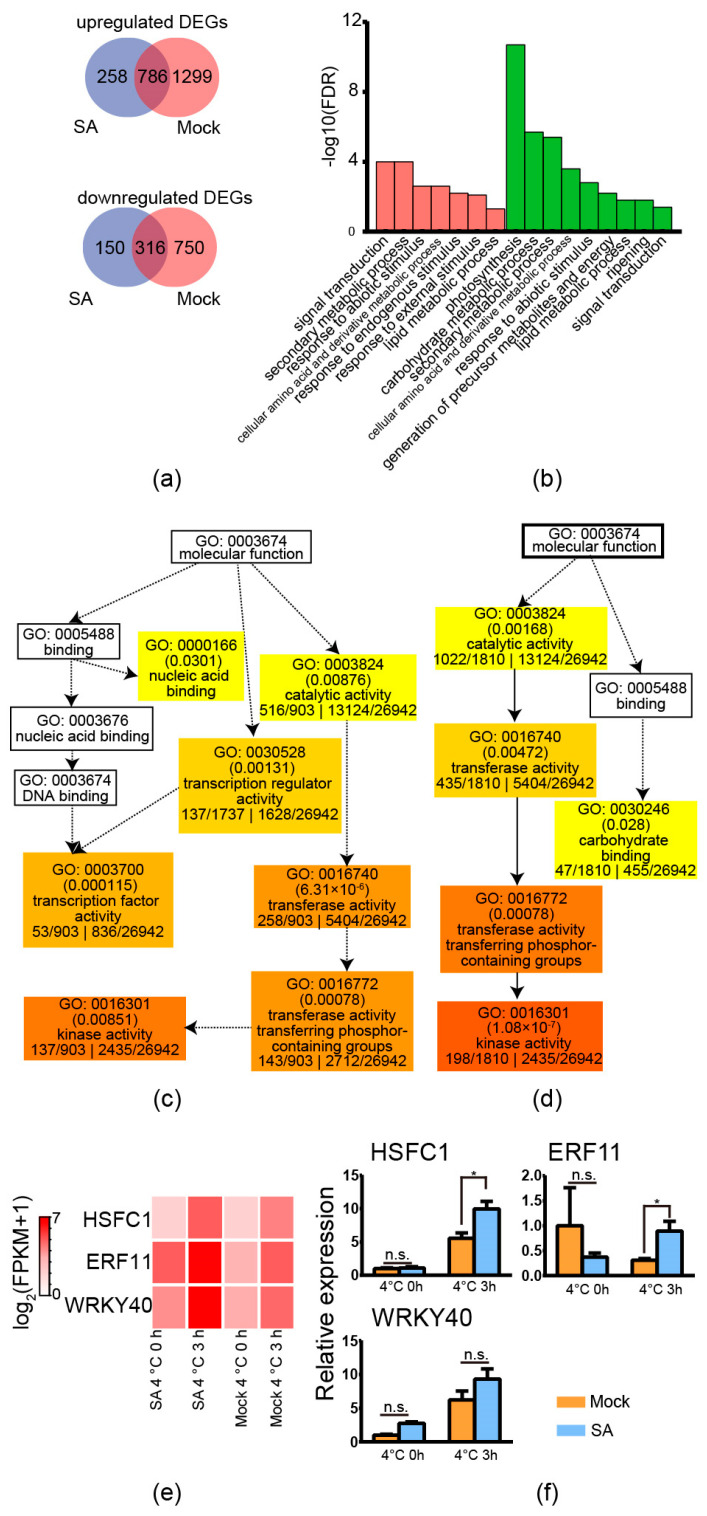
RNA-seq and qPCR detection of tub with exogenous application of SA and distilled water. (**a**) Venn diagram of the upregulated and downregulated DEGs of tub with distilled water (mock) and tub with exogenous application of 0.1 mM SA after exposure to 4 °C for 3 h compared to the control condition. (**b**) Enriched GO “biological process” terms in the upregulated DEGs of mock treatment and SA treatment after exposure to 4 °C for 3 h compared to the control condition, respectively. The red bars indicate the SA treatment after exposure to 4 °C for 3 h compared to the control condition. The green bars indicate the mock treatment after exposure to 4 °C for 3 h compared to the control condition. (**c**) Directed acyclic graph of GO “molecular function” terms of upregulated DEGs of the SA treatment after exposure to 4 °C for 3 h compared to the control condition. (**d**) Directed acyclic graph of GO “molecular function” terms of upregulated DEGs of the mock after exposure to 4 °C for 3 h compared to the control condition. (**e**) Expression patterns of *HSFC1*, *ERF11,* and *WRKY 40* of the mock and SA treatment after exposure to 4 °C for 3 h compared to the control condition derived from RNA-seq. (**f**) qPCR analysis of the expression patterns of *HSFC1*, *ERF11,* and *WRKY 40* of the mock and SA treatment after exposure to 4 °C for 3 h compared to the control condition. Asterisks indicate significant differences detected by Student’s *t*-test, *p* < 0.05. n.s.: no significance.

To analyse the downstream genes of exogenous application of SA to increase freezing tolerance, RNA-seq was applied. According to our previous research, the treatment of 4 °C for 3 h could represent whether the downstream *CBFs* have been activated [11]. Therefore, three independent biological replicates of exogenous application of SA and distilled water (mock) on tub before/after exposure to 4 °C for 3 h were used for RNA-seq analysis. Leaf samples of four-week-old tub plantlets were used for RNA-seq. Each biological replicate was pooled by three technical replicates. In each technical replicate, five leaves of a plantlet were used. As shown in Figure 6a,b, there were 258 specifically upregulated genes and 150 specifically downregulated genes in tub with exogenous application of SA compared with the mock after exposure to cold conditions. The absolute value of the log2 (fold change with FPKM) ≥1 and FDR ≤0.001 between exogenous application of SA and mock was used to determine the DEGs. Further GO “biological process” enrichment analysis showed that “response to endogenous stimulus” and “response to external stimulus” were specifically enriched in tub by the exogenous application of SA (Figure 6c). The enriched GO terms might be caused by exogenously-applied SA. Moreover, the results of GO “molecular function” analysis showed that GO: 0003700 “transcription factor activity” was specifically enriched in tub by exogenous application of SA. This result suggested that the exogenous application of SA activated transcription factors, which might be related to freezing tolerance. The transcription factors included the non-CBF transcriptional factors *HSFC1*, *ERF11,* and *WRKY40,* but not *CBF* members (Supplementary Table S4). The heatmap derived from RNA-seq showed that the expression levels of *HSFC1*, *ERF11,* and *WRKY40* were upregulated by the exogenous application of SA after exposure to cold conditions compared with the control condition (Figure 6c). QPCR results supported that *HSFC1* and *ERF11* were specifically upregulated in tub by the exogenous application of SA compared with the mock (significant difference as indicated by independent *t*-test analysis with *p* ≤ 0.05). These results suggested that the exogenous application of SA possibly improved freezing tolerance via non-CBF members, such as *HSFC1*.

## 3. Discussion

Deciphering the molecular processes of cold hardiness in freezing-tolerant wild species, such as *S. acaule*, is beneficial to innovate cultivation and speed up potato breeding against hardiness. In the present study, SA was documented to play a role in the freezing tolerance of *S. acaule*. The content of SA increased in aca after exposure to non-acclimated cold conditions. In addition to in potato, the content of SA increases in many species after exposure to cold conditions, such as *Arabidopsis*, wheat, grape berry, and cucumber [28,29,30,31]. This suggests that the accumulation of endogenous SA after exposure to cold conditions is a common strategy for plants in response to cold conditions. When the accumulation of endogenous SA is inhibited, such as in cucumber, freezing tolerance is reduced [31]. This suggests that the accumulation of endogenous SA plays an important role in freezing tolerance of plants.

The effect of SA on freezing tolerance was previously shown to be related to the modulation of H_2_O_2_ content, such as in cucumber and *Arabidopsis* [32,33]. However, in potato, Mora-Herrera et al. [18] indicated that SA and H_2_O_2_ improve freezing tolerance by independent pathways. This suggests that the underlying mechanism of SA on freezing tolerance is different from those of the above-mentioned species. The CBF pathway is the central regulator of the freezing tolerance of plants. Therefore, we subsequently analysed the expression of *CBF* in tub with the exogenous application of SA. Intriguingly, the exogenous application of SA did not promote the expression of *CBF*. We speculated that the exogenous application of SA might increase freezing tolerance via the non-CBF pathway. As CBFs only regulate 10–25% of cold-responsive genes [33], many *COR* genes are regulated by non-CBF genes, such as *CRY2*-*COP1*-*HY5* [34]. Since the GO term “transcription factor activity” was specifically enriched in the exogenous application of SA compared with the mock treatment (Figure 6c), the transcription factor might contribute to the freezing tolerance of SA. In the research of Park et al. [23], 27 other “first-wave” transcription factors that potentially regulate freezing tolerance, including *CBF*, were identified. Five non-CBF transcription factors, including *HSFC1*, were shown to regulate *COR* genes. In the present research, exogenous application of SA was shown to promote the expression of *HSFC1*; therefore, we indicated that SA increases the freezing tolerance of potato, possibly via non-CBF transcription factors such as *HSFC1*.

In addition to SA, putrescine, and its synthesis enzyme ADC1, were also proven to regulate freezing tolerance of *S. acaule* in our previous research [13]. Moreover, we also identified the MKK2, a member of the mitogen-activated protein kinase (MAPK) signalling pathway and a calcineurin B-like gene, member of the calcium signaling pathway, which contributed to freezing tolerance of *S. acaule* [10,11]. This implied that at least the mentioned pathways participate in the freezing tolerance of *S. acaule*. In fact, Thomashow [8] indicated that freezing tolerance of plants was determined by a quantitative trait locus. Many genes are supposed to contribute to freezing tolerance of plants. Therefore, it would be valuable to decipher more pathways, genes, and metabolites in addition to the mentioned ones that determine freezing tolerance of *S. acaule*.

The role of SA in potato freezing tolerance was revealed by comparative transcriptomics and metabolomics analysis in the present study. The combination of transcriptomics and metabolomics analysis was also successfully used to unravel the putrescine pathway that regulates potato freezing tolerance [13]. Raja et al. [12] also revealed the pathways for cold tolerance in rapeseed by this method. This result suggested that the combination of transcriptomics and metabolomics analyses is a useful method for uncovering the pathways and metabolites that regulate freezing tolerance.

There are at least two methods to combine transcriptomics and metabolomics data. The first method is the combination of the DEGs and differentially-accumulated metabolites according to the known metabolic pathways, such as the KEGG pathways. We used this method to identify putrescine and SA in our previous research and the present study, respectively [13]. The method only selects the pathways in which the expression of genes and the content of metabolites are varied at the same time. This approach is accurate but depends on the information of the existing pathway. Most of the time, the genes identified by this method are metabolite metabolism-related genes, such as the putrescine synthesis gene *ADC1* in our previous research. The second method is to combine the DEGs with differentially-accumulated metabolites by correlation network analysis. Raze et al. [12] and Jin et al. [35] successfully used this method to construct interaction networks between cold-regulated genes and metabolites in rapeseed and tobacco, respectively. Because the method is independent of the existing pathway, some potential relationships between the genes and metabolites, such as that transcription factors regulate metabolites’ metabolism, can be unraveled. Since the correlation analysis is bidirectional, it might be difficult to confirm whether the gene regulates the content of the metabolites or whether the metabolite regulates the expression of the genes. The first method was used in the present study.

In addition to SA revealed by the combination of transcriptomics and metabolomics analysis, many genes and metabolites were also activated when the two genotypes were transferred to –1 °C for 12 h. The KEGG pathways “arginine and proline metabolism” were enriched in the upregulated DEGs between aca and tub under –1 °C for 12 h. The DEGs of the pathway contained synthesis genes related to agmatine, 4-amino butanoate, spermidine, putrescine, glutamate, and 1-pynoline 5-carboxylate. Among the activated metabolites, putrescine was identified in further metabolomics assays (Figure 4), and our previous research also documented the roles of putrescine and its synthesis enzyme ADC1 in regulating freezing tolerance in potato [13]. Our previous research also showed that the content of spermidine before/after exposure to cold conditions in potato was not significantly changed [13]. Because the metabolomics assay carried out in the present study did not cover agmatine, 4-amino butanoate, glutamate, and 1-pynoline 5-carboxylate, whether these metabolites are related to freezing tolerance of potato is unknown. Some of the metabolites, such as agmatine, were identified to be involved in *Pringlea antiscorbutica* in adaptation to cold environments [20]. It will be interesting to decipher the roles of the metabolites in future research.

## 4. Materials and Methods

### 4.1. Plant Material and Measurement of Freezing Tolerance

In vitro plantlets of freezing-tolerant *S. acaule* ACL-27 (aca) and freezing-sensitive *S. tuberosum* 10908-06 (tub) were cultured at 20 ± 1 °C on MS (Murashige and Skoog) medium supplemented with 4% sucrose and 0.7% agar in a culture room under a light intensity of 60 μmol·m^–2^·s^–1^. Four-week in vitro plantlets were transplanted into pots and cultured in a greenhouse at 22 ± 2 °C with a 16 h photoperiod at Huazhong Agricultural University, Wuhan, China, with a light intensity of 250 μmol/m^2^/s. Four-week-old plantlets were used for measurement of freezing tolerance and cold treatment.

The measurement was carried out by electrolyte leakage of the excised leaflets and the phenotype of freezing damage of the whole plantlets before/after being transferred to cold conditions. The electrolyte leakage of the excised leaflets was measured according to our previous research [13]. The freezing damage of the whole plantlets before/after being transferred to cold conditions was conducted in accordance with our previous research [10]. Briefly, four-week-old plantlets were treated with or without cold acclimation at 4 °C for seven days and then transferred into a low-temperature growth chamber. The plants without cold acclimation were kept at −3.0 °C for six hours, followed by recovery at an appropriate temperature for two days. The plants subjected to cold acclimation were subjected to −3.5 °C for six hours, followed by the same recovery procedure. The photoperiod was maintained at 16 h light/8 h dark during cold conditions. Three independent assays were performed, and representative plants were photographed.

### 4.2. Freezing Stress Treatment for Transcriptomics and Metabolomics Analysis

For freezing stress treatment, uniform-sized plants were selected to be transferred into a controlled chamber (Conviron S10H, Winnipeg, Canada) with a 16 h light/8 h dark photoperiod at 4 °C for 7 d and then transferred to –1 °C for another 12 h with the same photoperiod. The plantlets of the different genotypes or treatments were randomly placed in the chamber in each biological repeat to avoid the possibility that the cold temperatures of the chamber were nonuniform. This freezing treatment regime was referred to as the acclimated freezing treatment, which was applied to differentiate it from the non-acclimated freezing treatment used in our previous research [13]. In the previous study, freezing treatment was applied without prior acclimation at 4 °C, i.e., four-week-old plants were transferred to –1 °C for 12 h without prior acclimation. Because potato growth ceases during freezing treatment [3], the acclimated and non-acclimated freezing treatments are regarded as having the same developmental state. The light intensity of 4 °C acclimation is 80 μmol/m^2^/s. The light was completely dark when plantlets were transferred into –1 °C. The sampling times were set consistent with our previous study [13]. The mature leaves from the second to fourth layers were collected, immediately frozen in liquid nitrogen, and stored at 80 °C prior to use. The abbreviations aca 4 °C 7 d –1 °C 12 h and tub 4 °C 7 d –1 °C 12 h were used as the abbreviations for aca and tub after exposure to 4 °C for 7 d with additional –1 °C for 12 h. The abbreviations of aca –1 °C for 12 h and tub –1 °C for 12 h were used as abbreviations for aca and tub after exposure to –1 °C for 12 h.

### 4.3. Transcriptome Sequencing and Data Analysis

Five independent replicates of each treatment were collected, three of which were randomly selected and used for RNA-seq analysis. The total RNA of the samples was extracted using TRIzol (Invitrogen, Carlsbad, CA, USA) and the remaining DNA was removed by digestion with DNase I (Fermentas, Waltham, MA, USA). Only intact RNA with an optical density at 260 nm/optical density at 280 nm was used. Oligo (dT) magnetic beads were applied to collect the mRNA from the total RNA. The mRNA was then fragmented into short fragments (approximately 200 bp) by using fragmentation buffers. The mRNA fragments were used as templates to synthesize the first strand of cDNA by random hexamer primers. The second strand was then synthesized. The double-strand of cDNA was purified with a QiaQuick PCR extraction kit (Qiagen, Hilden, Germany) according to the manufacturer’s instructions and was washed with EB buffer for end repair and poly-A addition. Finally, the fragments were ligated to sequencing adaptors, purified by agarose gel electrophoresis, and enriched by PCR amplification for library construction. The library products were sequenced using an Illumina HiSeqTM 2000 (San Diego, CA, USA).

Raw reads were filtered and mapped as described previously [13]. Cuffdiff was used to analyse DEGs (differentially expressed genes) of the genotypes under –1 °C for 12 h and 4 °C for 7 d with additional –1 °C for 12 h [36]. Briefly, a threshold to determine the absolute value of the log2 (fold change with FPKM, fragments per kb per million reads) ≥ 1 and false discovery rate (FDR) ≤ 0.001 was used to delineate significant differences of the gene expression. Gene Ontology (GO) enrichment analyses were carried out using agriGO [37]. Kyoto Encyclopedia of Genes and Genomes (KEGG) enrichment analyses were performed with custom-developed Perl scripts. An FDR-corrected *p*-value ≤ 0.05 was used as a cut-off value for the significance of both GO and KEGG enrichment analyses. The raw data were deposited in the National Center for Biotechnology Information with the accession number PRJNA593373.

### 4.4. Quantitative Real-Time Reverse Transcription Polymerase Chain Reaction (qRT-PCR)

Samples were the same for RNA-seq experiments. cDNA synthesis and qRT-PCR were carried out as described [13]. The primers used for gene detection are listed in Supplementary Table S1. Endogenous ef1α was chosen to calibrate the expression level of the query genes, as it is an appropriate reference gene for freezing stress in potato [38]. The 2^−ΔCt^ method was applied to calculate the relative expression levels of target genes. For each gene, three biological replicates with tri-replications were included.

### 4.5. Metabolite Profiling

Samples were collected as described in 4.1. Five independent replicates of each treatment were used for metabolic profiling. Metabolites, except abscisic acid (ABA) and SA, were profiled using GC-MS as previously described [13]. The ABA content was measured by the Plant ABA Enzyme-linked Immunosorbent Assay (ELISA) Kit (AB-3466A, Abmart, Shanghai, China) according to the manufacturer’s instructions. The SA content was measured by the Plant SA ELISA Kit (AB-3468A, Abmart, Shanghai, China) according to the manufacturer’s instructions. Metabolites were classed into three types according to KEGG annotation. Type 1 is the “amino acid-related pathways” including the metabolites belonging to the KEGG pathways “biosynthesis of amino acids”, “arginine and proline metabolism”, and “phenylalanine metabolism”. Type 2 is “saccharide-related pathway”, comprising the metabolites of the KEGG pathways “pentose phosphate pathway”, “starch and sucrose metabolism”, “galactose metabolism”, and “amino sugar and nucleotide sugar metabolism”. Type 3 is “other metabolites-related pathways” and contains metabolites of other KEGG pathways except the mentioned pathways.

### 4.6. Exogenous Application of SA

Four-week-old plantlets of the freezing-sensitive genotype tub were used. Exogenous application of SA was conducted according to Li et al. [39]. The plantlets were sprayed with 0.01 mM and 0.1 mM SA. The mock was sprayed with only water. All the leaves were sprayed until completely wet. The measurement of freezing tolerance was carried out after 24 h.

### 4.7. Statics Analysis

DEGs of RNA-seq were calculated by Cuffdiff. An absolute value of log2 (fold change with FPKM) ≥1 and false discovery rate (FDR) ≤0.001 were used to indicate significant differences. Gene Ontology (GO) enrichment analyses were carried out using agriGO [37]. Kyoto Encyclopedia of Genes and Genomes (KEGG) enrichment analyses were performed with custom-developed Perl scripts. An FDR-corrected *p* value ≤ 0.05 was used as a cut-off value for the significance of both GO and KEGG enrichment analyses. SPSS 20 was used to analyse and determine the statistical significance of the metabolomics, qRT-PCR, agricultural traits, and electrolyte leakage measurement results based on two-tailed independent Student’s *t*-tests. *p* < 0.05 was considered statistically significant.

## 5. Conclusions

In summary, to identify the potential pathways and metabolites that regulate the freezing tolerance of potato, comparative transcriptomic and metabolomic analyses were carried out on a freezing-tolerant potato genotype (aca) and a freezing-sensitive genotype (tub). SA was documented to play a role in the improvement of potato freezing tolerance. The underlying mechanism of SA in enhancing freezing tolerance was also primarily uncovered. Our study provides novel information on SA, indicating that it regulates the constitutive freezing tolerance of potato, enriching our understanding of potato freezing tolerance that is mediated by hormones.

## Figures and Tables

**Table 1:** The plant height, stem diameter, and leaf area of four-week-old aca and tub ^a^.

	Plant Height (cm)	Stem Diameter (mm)	Leaf Area (cm^2^)
aca	24.3 ± 2.0	4.6 ± 0.8	4.1 ± 0.5
tub	34.1 ± 1.7	7.9 ± 0.4	6.8 ± 0.7
*p*	<0.001	<0.001	<0.001

^a^ Data are presented as mean ± S.E. of five replicates. *p* value indicates a significant difference between aca and tub, analyzed by independent samples *t*-test.

## Data Availability

The raw reads of RNA-seq were deposited into the National Center for Biotechnology Information Sequence Reads Archive under the accession number PRJNA793753.

## Supplementary Materials

The supporting information can be downloaded at: https://www.mdpi.com/article/10.3390/ijms24010609/s1.
