# Supplementary material for: Salicylic Acid Improves the Constitutive Freezing Tolerance of Potato as Revealed by Transcriptomics and Metabolomics Analyses
Source: Int J Mol Sci. 2022 Dec 29;24(1):609. doi: 10.3390/ijms24010609 (PMC9820103; doi:10.3390/ijms24010609)
Supplement: Supplementary file 1 [file ijms-24-00609-s001.zip › ijms-2059135-Supplementary_Material.pdf]

Supplementary Figures

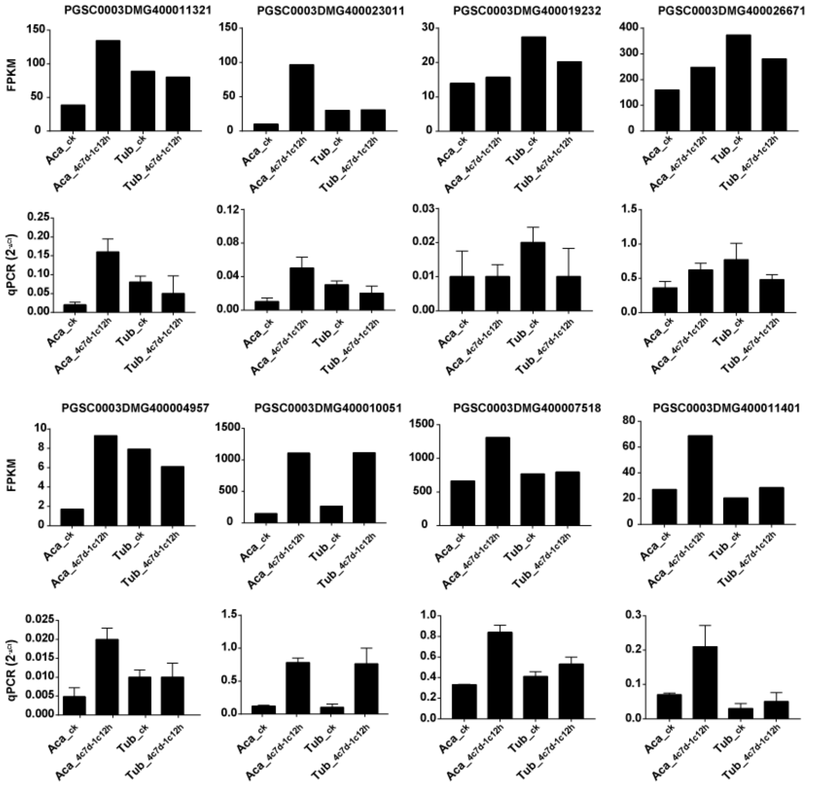

(a)

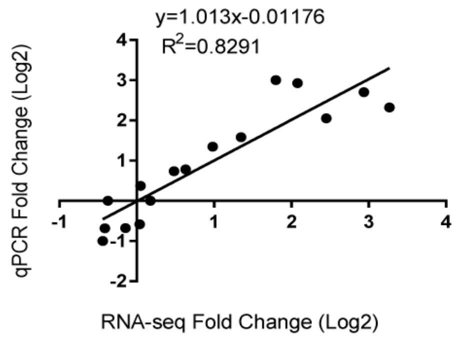

(b)

Figure S1. Verification of gene expression by qRT-PCR. Eight randomly selected genes are used for the analysis. FPKM: FPKM is derived by mapping RNA-seq reads to reference genome and calculating the value with cufflinks; qRT-PCR, the expression of each gene that was calculated as the $2^{-\Delta Ct}$ value and normalized to the endogenous reference gene ef1α. QPCR data are showed as mean ± S.D. of three biological replicates.

Supplementary Figure S2: The enriched KEGG pathway "Arginine and proline metabolism"of up-regulated DEGs of comparison between aca and tub under –1 °C for 12 h. The pink color indicated the DEGs.

Supplementary Figure S3: The enriched KEGG pathway "Tyrosine metabolism"of down-regulated DEGs of comparison between aca and tub under 7 d4°C for 7 d –1 °C for 12 h. The pink color indicated the DEGs.

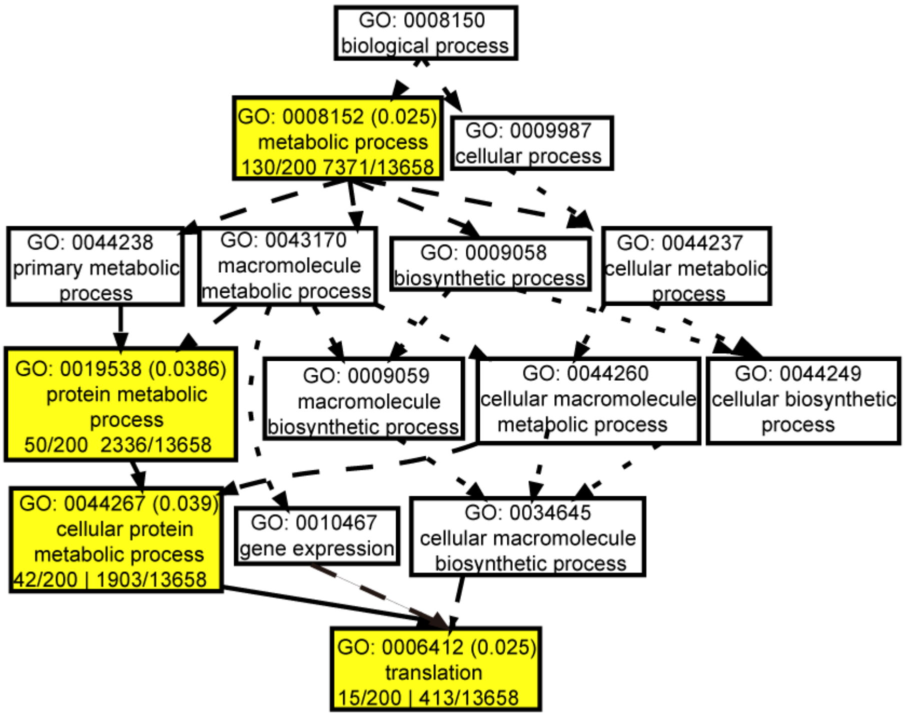

Figure S4 Enriched GO pathways of the overlapping DEGs of aca in response to both two types of cold conditions compared with the control condition.

Supplementary Tables

Table S1. Forward and reverse primer sequences of the genes used in qRT-PCR

Gene code	Forward primer	Reverse primer
PGSC0003DMG400011321	TGGCTATAACTTCACAGATG	AGGAATTGTTGGCTCTATG
PGSC0003DMG400023011	GTGTTGATGGAATGATAAGC	GCATACCAAGCGTTTACTA
PGSC0003DMG400019232	ACGCTATGACTACTACTC	CCAGGCTATCACTTAATG
PGSC0003DMG400011401	GATGGCAATAGGAAGAAGA	CAAACAGAAGGCTGAATG
PGSC0003DMG400004957	TTGTGATGTCGGCTAACG	GTGTTGTACGCTAACTTCCA
PGSC0003DMG400026671	CATGCCTTATCTTGCGCCAC	CCTATTGCACATTCTGCGCC
PGSC0003DMG400010051	AAACTGCTGCTCTCGATCCC	AAGTGGCGATGTGGGTATGG
PGSC0003DMG400007518	TTTCTTTCGTCGAGCCTGGT	CACTCAGCTGGTCCGAGAAT
PGSC0003DMG400014310 (CBF1)	CGTACTCAGTATTTGGATGGTACG	TTCCTCTCACTGCCCAAACC
PGSC0003DMG400000380 (HSFC1)	TGCGATCTGTCAGTCCTCTC	TTCGCGTCAGGAGATCGAAT
PGSC0003DMG400022823 (ERF11)	CGATGAATTACCGAAACAGGAAGT	TCTTCGATCCATCGCTGTTCT
PGSC0003DMG400019824 (WRKY40)	CTGTTGAATCGTCGAGTCCG	TGGTTAAACCCGCCGATTG

Table S2. Summary of transcriptome sequencing data

Samples[1]	Number of raw reads	Number of clean reads (%[2])	Number of mapped reads (%[3])	Q30(%)
Tub 4 ºC 7d -1 ºC 12h_rep1	10,053,630	8,867,562 (88.2)	6,943,301 (78.3)	94.0
Tub 4 ºC 7d -1 ºC 12h_rep2	10,378,798	9,115,858 (87.8)	7,520,583 (82.5)	94.5
Tub 4 ºC 7d -1 ºC 12h_rep3	11,448,922	10,212,698 (89.2)	8,609,304 (84.3)	94.6
Aca 4 ºC 7d -1 ºC 12h_rep1	9,718,730	9,439,726 (97.1)	7,655,618 (81.1)	94.8
Aca 4 ºC 7d -1 ºC 12h_rep2	10,432,182	10,122,340 (97.0)	8,178,851 (80.8)	94.5
Aca 4 ºC 7d -1 ºC 12h_rep3	10,780,568	10,368,478 (96.2)	8,325,888 (80.3)	94.8
Average	10,468,805	9,687,777 (92.5)	7,872,257 (81.3)	94.5

[1] Tub 4 ºC 7d -1 ºC 12h and Aca 4 ºC 7d -1 ºC 12h indicate samples of *Tub* and *Aca* exposured to 4 °C for seven days and the following -1 °C for 12h, respectively

[2] percentage = number of clean reads / number of all reads × 100

[3] percentage = number of mapped reads / number of clean reads × 100

Table S3. The raw data of GC-MS and enzyme linked immunosorbent assay evaluated metabolites

Table S4. Gene list of the enriched GO molecular function term "transcription activity" of tub with exogenous application of SA after exposure to cold condition comparing with control condition

Gene code	Gene annotation	GO annotation
Soltu.DM.12 G024950	HSFC1	GO:0003700 transcription factor activity<br>GO:0043565 sequence-specific DNA binding<br>GO:0005634 nucleus<br>GO:0042025 host cell nucleus
Soltu.DM.10 G005000	ERF11	GO:0003700 transcription factor activity<br>GO:0005634 nucleus<br>GO:0003677 DNA binding
Soltu.DM.03 G030960	WRKY40	GO:0003700 transcription factor activity<br>GO:0043565 sequence-specific DNA binding<br>GO:0005634 nucleus
Soltu.DM.05 G026450	WRKY DNA-binding protein	GO:0003700 transcription factor activity<br>GO:0043565 sequence-specific DNA binding<br>GO:0005634 nucleus
Soltu.DM.01 G041210	myb domain protein	GO:0044212 DNA regulatory region binding<br>GO:0043565 sequence-specific DNA binding<br>GO:0003700 transcription factor activity<br>GO:0005634 nucleus<br>GO:0030154 cell differentiation<br>GO:0010119 regulation of stomatal movement<br>GO:0003677 DNA binding

Soltu.DM.02 G031730	myb domain protein	GO:0009733 response to auxin stimulus<br>GO:0044212 DNA regulatory region binding<br>GO:0009617 response to bacterium<br>GO:0043565 sequence-specific DNA binding<br>GO:0003700 transcription factor activity<br>GO:0005634 nucleus<br>GO:0030154 cell differentiation<br>GO:0009626 plant-type hypersensitive response<br>GO:0003677 DNA binding<br>GO:0001666 response to hypoxia<br>GO:0042761 very-long-chain fatty acid biosynthetic process
Soltu.DM.12 G007270	bZIP transcription factor family protein	GO:0003700 transcription factor activity<br>GO:0043565 sequence-specific DNA binding<br>GO:0006351 transcription, DNA-dependent
Soltu.DM.06 G028040	sigma factor A	GO:0003700 transcription factor activity<br>GO:0006352 transcription initiation
Soltu.DM.06 G009910	HXXXD-type acyl-transferase family protein	GO:0003700 transcription factor activity<br>GO:0005634 nucleus<br>GO:0016747 transferase activity, transferring acyl groups other than amino-acyl groups<br>GO:0003677 DNA binding
Soltu.DM.04 G029170	related to AP2	GO:0003700 transcription factor activity<br>GO:0043565 sequence-specific DNA binding<br>GO:0005634 nucleus<br>GO:0003677 DNA binding
Soltu.DM.11 G025890	related to AP2.7	GO:0003700 transcription factor activity<br>GO:0005634 nucleus<br>GO:0003677 DNA binding

Soltu.DM.08 G015040	Integrase-type DNA-binding superfamily protein	GO:0003700 transcription factor activity<br>GO:0005634 nucleus<br>GO:0003677 DNA binding
Soltu.DM.03 G030200	myb domain protein	GO:0009733 response to auxin stimulus<br>GO:0044212 DNA regulatory region binding<br>GO:0043565 sequence-specific DNA binding<br>GO:0003700 transcription factor activity<br>GO:0005634 nucleus<br>GO:0030154 cell differentiation<br>GO:0003677 DNA binding
Soltu.DM.07 G023850	TCP family transcription factor	GO:0003700 transcription factor activity<br>GO:0043565 sequence-specific DNA binding<br>GO:0005634 nucleus<br>GO:0048831 regulation of shoot development
Soltu.DM.04 G000120	myb-like transcription factor family protein	GO:0003700 transcription factor activity<br>GO:0005634 nucleus<br>GO:0008270 zinc ion binding<br>GO:0003677 DNA binding<br>GO:0003682 chromatin binding<br>GO:0031540 regulation of anthocyanin biosynthetic process
Soltu.DM.08 G002290	WRKY DNA-binding protein	GO:0003700 transcription factor activity<br>GO:0043565 sequence-specific DNA binding<br>GO:0005634 nucleus
Soltu.DM.06 G020280	TCP family transcription factor	GO:0003700 transcription factor activity<br>GO:0043565 sequence-specific DNA binding<br>GO:0005634 nucleus

Soltu.DM.06 G025970	myb domain protein	GO:0009733 response to auxin stimulus<br>GO:0044212 DNA regulatory region binding<br>GO:0043565 sequence-specific DNA binding<br>GO:0003700 transcription factor activity<br>GO:0005634 nucleus<br>GO:0030154 cell differentiation<br>GO:0003677 DNA binding
Soltu.DM.08 G004690	WRKY family transcription factor	GO:0003700 transcription factor activity<br>GO:0044212 DNA regulatory region binding<br>GO:0005634 nucleus<br>GO:0043565 sequence-specific DNA binding
Soltu.DM.05 G003020	Integrase-type DNA-binding superfamily protein	GO:0003700 transcription factor activity<br>GO:0005634 nucleus<br>GO:0003677 DNA binding
Soltu.DM.10 G024000	C2H2-like zinc finger protein	GO:0003700 transcription factor activity<br>GO:0005634 nucleus<br>GO:0046872 metal ion binding<br>GO:0003676 nucleic acid binding
Soltu.DM.03 G022850	sigma factor E	GO:0003700 transcription factor activity<br>GO:0006352 transcription initiation
Soltu.DM.02 G025460	GRAS family transcription factor	GO:0043565 sequence-specific DNA binding<br>GO:0003700 transcription factor activity<br>GO:0005634 nucleus<br>GO:0030154 cell differentiation<br>GO:0006355 regulation of transcription, DNA-dependent<br>GO:0048831 regulation of shoot development

Soltu.DM.02 G031630	cycling DOF factor	GO:0003700 transcription factor activity<br>GO:0006355 regulation of transcription, DNA-dependent<br>GO:0005634 nucleus<br>GO:0003677 DNA binding
Soltu.DM.12 G022800	erf domain protein	GO:0003700 transcription factor activity<br>GO:0006952 defense response<br>GO:0005634 nucleus<br>GO:0009873 ethylene mediated signaling pathway<br>GO:0003677 DNA binding
Soltu.DM.02 G027840	GRAS family transcription factor	GO:0003700 transcription factor activity<br>GO:0006355 regulation of transcription, DNA-dependent<br>GO:0005634 nucleus<br>GO:0043565 sequence-specific DNA binding
Soltu.DM.03 G027860	Calmodulin binding protein-like	GO:0003700 transcription factor activity<br>GO:0043565 sequence-specific DNA binding<br>GO:0005634 nucleus<br>GO:0005516 calmodulin binding<br>GO:0080142 regulation of salicylic acid biosynthetic process
Soltu.DM.10 G023760	WRKY DNA-binding protein	GO:0003700 transcription factor activity<br>GO:0043565 sequence-specific DNA binding<br>GO:0005634 nucleus
Soltu.DM.02 G031540	myb domain protein	GO:0044212 DNA regulatory region binding<br>GO:0043565 sequence-specific DNA binding<br>GO:0003700 transcription factor activity<br>GO:0005634 nucleus<br>GO:0030154 cell differentiation<br>GO:0003677 DNA binding

Soltu.DM.09 G030640	basic helix-loop-helix (bHLH) DNA-binding superfamily protein	GO:0003700 transcription factor activity<br>GO:0006355 regulation of transcription, DNA-dependent<br>GO:0005634 nucleus<br>GO:0046983 protein dimerization activity
Soltu.DM.05 G019830	G-box binding factor	GO:0003700 transcription factor activity<br>GO:0006355 regulation of transcription, DNA-dependent<br>GO:0005634 nucleus<br>GO:0043565 sequence-specific DNA binding
Soltu.DM.08 G028970	Integrase-type DNA-binding superfamily protein	GO:0003700 transcription factor activity<br>GO:0005634 nucleus<br>GO:0003677 DNA binding
Soltu.DM.03 G029900	CCT motif -containing response regulator protein	GO:0007623 circadian rhythm<br>GO:0003700 transcription factor activity<br>GO:0000160 two-component signal transduction system (phosphorelay)<br>GO:0005634 nucleus<br>GO:0010629 negative regulation of gene expression<br>GO:0003677 DNA binding<br>GO:0010031 circumnutation
Soltu.DM.07 G025300	GRAS family transcription factor	GO:0003700 transcription factor activity<br>GO:0006355 regulation of transcription, DNA-dependent<br>GO:0005634 nucleus<br>GO:0043565 sequence-specific DNA binding
Soltu.DM.05 G005140	cycling DOF factor	GO:0003700 transcription factor activity<br>GO:0006355 regulation of transcription, DNA-dependent<br>GO:0005634 nucleus<br>GO:0003677 DNA binding
Soltu.DM.04 G035840	basic leucine-zipper	GO:0003700 transcription factor activity<br>GO:0044212 DNA regulatory region binding

		GO:0005634 nucleus<br>GO:0045893 positive regulation of transcription, DNA-dependent<br>GO:0043565 sequence-specific DNA binding
Soltu.DM.06 G030980	related to AP2.7	GO:0003700 transcription factor activity<br>GO:0005634 nucleus<br>GO:0003677 DNA binding
Soltu.DM.04 G019860	Integrase-type DNA-binding superfamily protein	GO:0044212 DNA regulatory region binding<br>GO:0043565 sequence-specific DNA binding<br>GO:0003700 transcription factor activity<br>GO:0005634 nucleus<br>GO:0045893 positive regulation of transcription, DNA-dependent GO:0003677 DNA binding
Soltu.DM.10 G003970	GOLDEN2-like / GBF's pro-rich region-interacting factor	GO:0003700 transcription factor activity<br>GO:0044212 DNA regulatory region binding<br>GO:0005634 nucleus<br>GO:0045893 positive regulation of transcription, DNA-dependent<br>GO:0003677 DNA binding
Soltu.DM.08 G011730	Basic-leucine zipper (bZIP) transcription factor family protein	GO:0010099 regulation of photomorphogenesis<br>GO:0009585 red, far-red light phototransduction<br>GO:0003700 transcription factor activity<br>GO:0005634 nucleus<br>GO:0010017 red or far-red light signaling pathway<br>GO:0010218 response to far red light<br>GO:0003677 DNA binding<br>GO:0010114 response to red light
Soltu.DM.10 G005890	WRKY family transcription factor	GO:0003700 transcription factor activity<br>GO:0044212 DNA regulatory region binding<br>GO:0005634 nucleus<br>GO:0043565 sequence-specific DNA binding

Soltu.DM.03 G033680	Calmodulin binding protein-like	GO:0003700 transcription factor activity<br>GO:0043565 sequence-specific DNA binding<br>GO:0005634 nucleus<br>GO:0005516 calmodulin binding<br>GO:0080142 regulation of salicylic acid biosynthetic process
Soltu.DM.08 G028690	cytokinin response factor	GO:0003700 transcription factor activity<br>GO:0005634 nucleus<br>GO:0003677 DNA binding
Soltu.DM.04 G023540	WRKY DNA-binding protein	GO:0003700 transcription factor activity<br>GO:0043565 sequence-specific DNA binding<br>GO:0005634 nucleus
Soltu.DM.12 G007120	related to AP2	GO:0003700 transcription factor activity<br>GO:0043565 sequence-specific DNA binding<br>GO:0005634 nucleus<br>GO:0003677 DNA binding
Soltu.DM.01 G034750	WRKY family transcription factor	GO:0003700 transcription factor activity<br>GO:0044212 DNA regulatory region binding<br>GO:0005634 nucleus<br>GO:0043565 sequence-specific DNA binding
Soltu.DM.07 G020230	GOLDEN2-like /GBF's pro-rich region-interacting factor	GO:0003700 transcription factor activity<br>GO:0044212 DNA regulatory region binding<br>GO:0005634 nucleus<br>GO:0045893 positive regulation of transcription, DNA-dependent<br>GO:0003677 DNA binding
Soltu.DM.01 G027400	nuclear factor Y, subunit A1	GO:0003700 transcription factor activity<br>GO:0006355 regulation of transcription, DNA-dependent<br>GO:0005634 nucleus

		GO:0016602 CCAAT-binding factor complex<br>GO:0003677 DNA binding
Soltu.DM.06 G026030	TEOSINTE BRANCHED, cycloidea and PCF (TCP)	GO:0003700 transcription factor activity<br>GO:0043565 sequence-specific DNA binding<br>GO:0005634 nucleus
Soltu.DM.01 G031210	Integrase-type DNA-binding superfamily protein	GO:0003700 transcription factor activity<br>GO:0005634 nucleus<br>GO:0003677 DNA binding
Soltu.DM.02 G009620	cycling DOF factor	GO:0003700 transcription factor activity<br>GO:0006355 regulation of transcription, DNA-dependent<br>GO:0005634 nucleus<br>GO:0003677 DNA binding
Soltu.DM.12 G012100	BR enhanced expression	GO:0003700 transcription factor activity<br>GO:0006355 regulation of transcription, DNA-dependent<br>GO:0005634 nucleus<br>GO:0046983 protein dimerization activity
Soltu.DM.07 G020090	erf domain protein	GO:0009536 plastid<br>GO:0003700 transcription factor activity<br>GO:0005634 nucleus<br>GO:0009873 ethylene mediated signaling pathway<br>GO:0006952 defense response<br>GO:0003677 DNA binding
