# Supplementary figures and images for: Salicylic Acid Improves the Constitutive Freezing Tolerance of Potato as Revealed by Transcriptomics and Metabolomics Analyses
Source: Int J Mol Sci. 2022 Dec 29;24(1):609. doi: 10.3390/ijms24010609 (PMC9820103; doi:10.3390/ijms24010609)

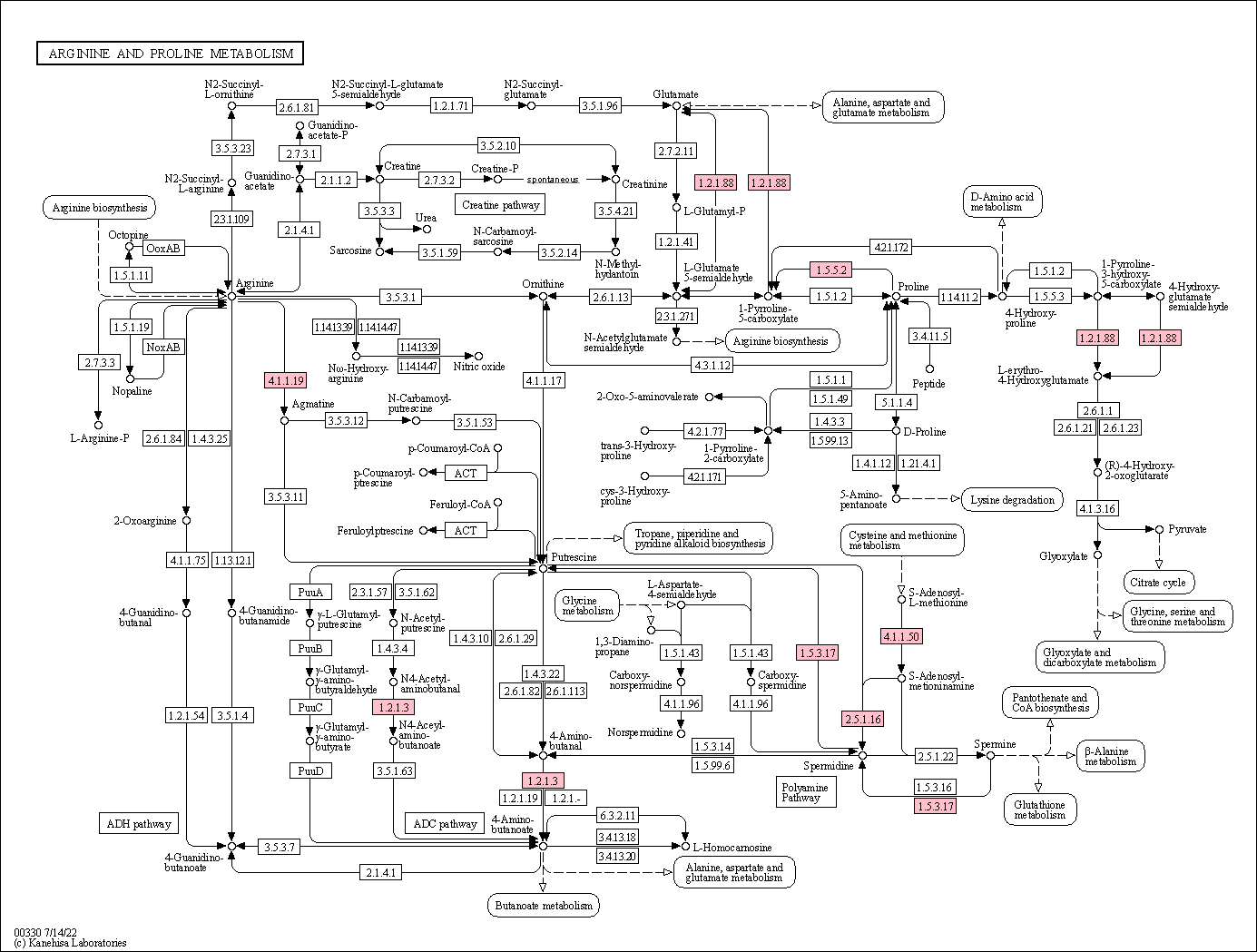

Supplement: Supplementary file 1 [file ijms-24-00609-s001.zip › Figure S2.png]

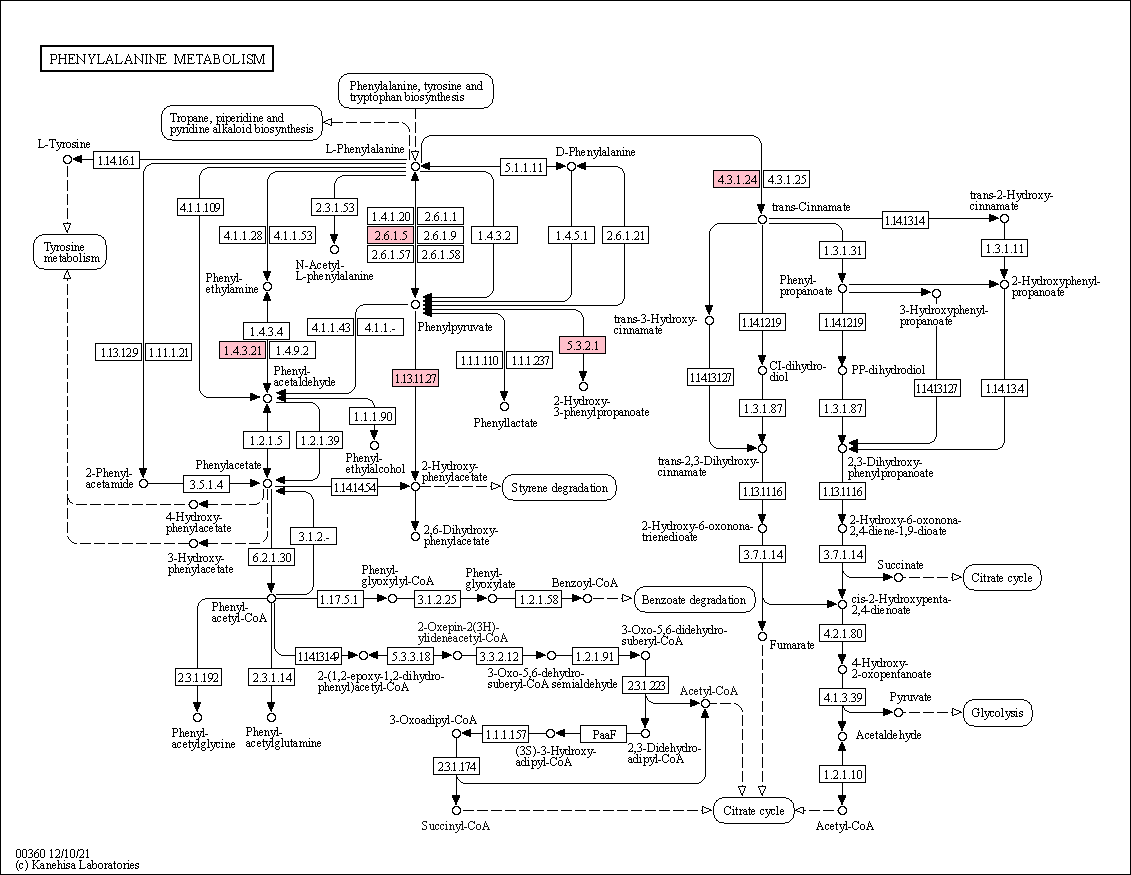

Supplement: Supplementary file 1 [file ijms-24-00609-s001.zip › Figure S3.png]
